# Hierarchical Multi‐Mode Computing in Interlayer‐Coupled 3D RRAM Crossbar Arrays

**DOI:** 10.1002/advs.76674

**Published:** 2026-07-30

**Authors:** Seungman Park, Jaewoo Choi, Gigon Nam, Junsu Yu, Donghyun Ryu, Jung‐Kyu Lee, Sungjoon Kim, Sungjun Kim

**Affiliations:** ^1^ Division of Electronics and Electrical Engineering Dongguk University Seoul Republic of Korea; ^2^ School of Electrical and Computer Engineering Georgia Institute of Technology Atlanta Georgia USA; ^3^ Electrical Engineering and Computer Sciences University of California Berkeley California USA; ^4^ Department of Semiconductor Engineering Gyeongsang National University Jinju Gyeongnam Republic of Korea; ^5^ Department of AI Semiconductor Engineering Korea University Sejong Republic of Korea

**Keywords:** 3D RRAM crossbar arrays, interlayer coupling, multimode computing, neuromorphic hardware, physical unclonable functions

## Abstract

This work presents a series‐stacked 2‐deck RRAM crossbar array with hierarchical multi‐mode operation enabled by a shared mid electrode. A 2 × 16 × 16 stacked crossbar array is fabricated, where the mid electrode electrically connects two vertically integrated resistive switching layers, simultaneously serving as the top electrode of the lower layer and the bottom electrode of the upper layer. This architecture enables bias‐selective access to independent single‐layer operation (1F and 2F) as well as electrically coupled serial operation (1F + 2F) within the same cell footprint. The shared mid electrode plays a key role in controlling voltage distribution and interlayer interaction, expanding the operational space beyond simple density scaling. Using an incremental step pulse with verify algorithm (ISPVA), all modes exhibit stable multilevel conductance modulation up to 6‐bit resolution (64 states) with clear state separation and retention exceeding 10^4^ s, while maintaining endurance over 100 switching cycles. System‐level evaluation using a VGG‐based CNN for CIFAR‐10 classification achieves inference accuracies of 93.37%, 93.38%, and 93.37% for the 1F, 2F, and 1F + 2F modes, respectively. The serial configuration also enables logic‐in‐memory functionality and demonstrates strong physical unclonable function (PUF) characteristics with near‐ideal uniformity (∼50%) and high entropy.

## Graphical abstract

2‐deck RRAM crossbar array provides a versatile hardware platform for multifunctional in‐memory computing. Stacked, series, and entropy node configurations enable multi‐layer conductance modulation, logic‐in‐memory operation, genetic learning, and reconfigurable physical unclonable functions. The resulting framework highlights how mid electrode engineering and 2‐deck integration can increase functional density while reducing architectural complexity.

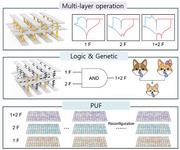

## 1 Introduction

Resistive random access memory has emerged as one of the most promising candidates for next‐generation nonvolatile memory and beyond‐von‐Neumann computing systems due to its simple metal–insulator–metal configuration, fast switching speed, scalability, and compatibility with back‐end‐of‐line integration [1, 2, 3, 4, 5, 6]. In crossbar architectures, RRAM devices can be directly arranged at the intersection of perpendicular metal lines, enabling ultrahigh‐density integration [7, 8, 9]. More importantly, the gradual modulation of device conductance allows resistive switching elements to emulate analog synaptic weights, making RRAM‐based arrays highly attractive for neuromorphic computing and in‐memory processing applications [10, 11, 12, 13, 14, 15, 16, 17]. As data‐centric workloads continue to expand, increasing memory density without enlarging the lateral footprint has become a critical challenge. 3D RRAM array architectures have therefore been widely investigated as a practical solution to enhance storage density and computational parallelism [18, 19, 20]. By integrating multiple switching layers along the vertical direction, 3D arrays offer improved bit density and potentially reduced interconnect length.

However, in many previously reported implementations, the stacked layers operate largely independently, functioning as replicated memory planes to achieve higher integration density [21]. In such cases, the vertical dimension primarily serves as a geometric scaling strategy rather than introducing fundamentally new device functionalities. Beyond density enhancement, multilayer integration offers an opportunity to engineer interlayer electrical interactions [22]. When two switching layers are electrically connected through a shared electrode or series configuration, the overall device behavior can no longer be described as a simple superposition of independent switching events. Instead, interlayer series coupling may lead to mode‐dependent threshold modulation, redistributed electric fields, and altered filament formation dynamics [23, 24]. These effects open a pathway toward multifunctional operation within a single‐cell footprint, where different electrical biasing schemes selectively activate individual layers or their combined response.

Despite this potential, systematic exploration of interlayer‐coupled 3D RRAM arrays for functional expansion rather than mere density scaling remains relatively limited. Simultaneously, emerging hardware systems increasingly demand the coexistence of computing capability and intrinsic security. RRAM devices inherently exhibit stochastic switching characteristics arising from nanoscale filament formation and rupture processes [25, 26, 27, 28]. While such variability can pose challenges for uniform memory operation, it can also serve as a valuable entropy source for hardware security primitives such as physical unclonable functions [29, 30, 31, 32, 33]. Integrating security functionality directly into memory or computing arrays eliminates the need for additional dedicated security hardware and reduces system complexity. However, achieving both reliable analog weight modulation for neuromorphic computing and sufficient randomness for security within a single architecture remains a nontrivial task.

In this work, we introduce a 3D dual‐layer RRAM crossbar array designed to exploit interlayer series coupling as a functional degree of freedom. The proposed architecture consists of two resistive switching stacks interconnected through an intermediate electrode, enabling distinct operational modes corresponding to individual layer operation and combined layer operation within the same cell area. Unlike conventional multilayer arrays that simply duplicate switching elements to increase density, the present structure intentionally leverages interlayer electrical interaction to modulate switching characteristics and expand the operational space. By systematically comparing the electrical behavior of each individual layer and their series‐coupled configuration, we demonstrate that the combined operation exhibits distinct switching dynamics and conductance modulation characteristics. This mode of reconfigurable behavior provides a unified platform capable of supporting multilevel analog weight control for neuromorphic computing while simultaneously offering an additional dimension for hardware security implementation. The results highlight that 3D RRAM integration can be engineered not only for density scaling but also for functional diversification through controlled interlayer coupling.

## 2 Results and Discussion

**FIGURE 1:**
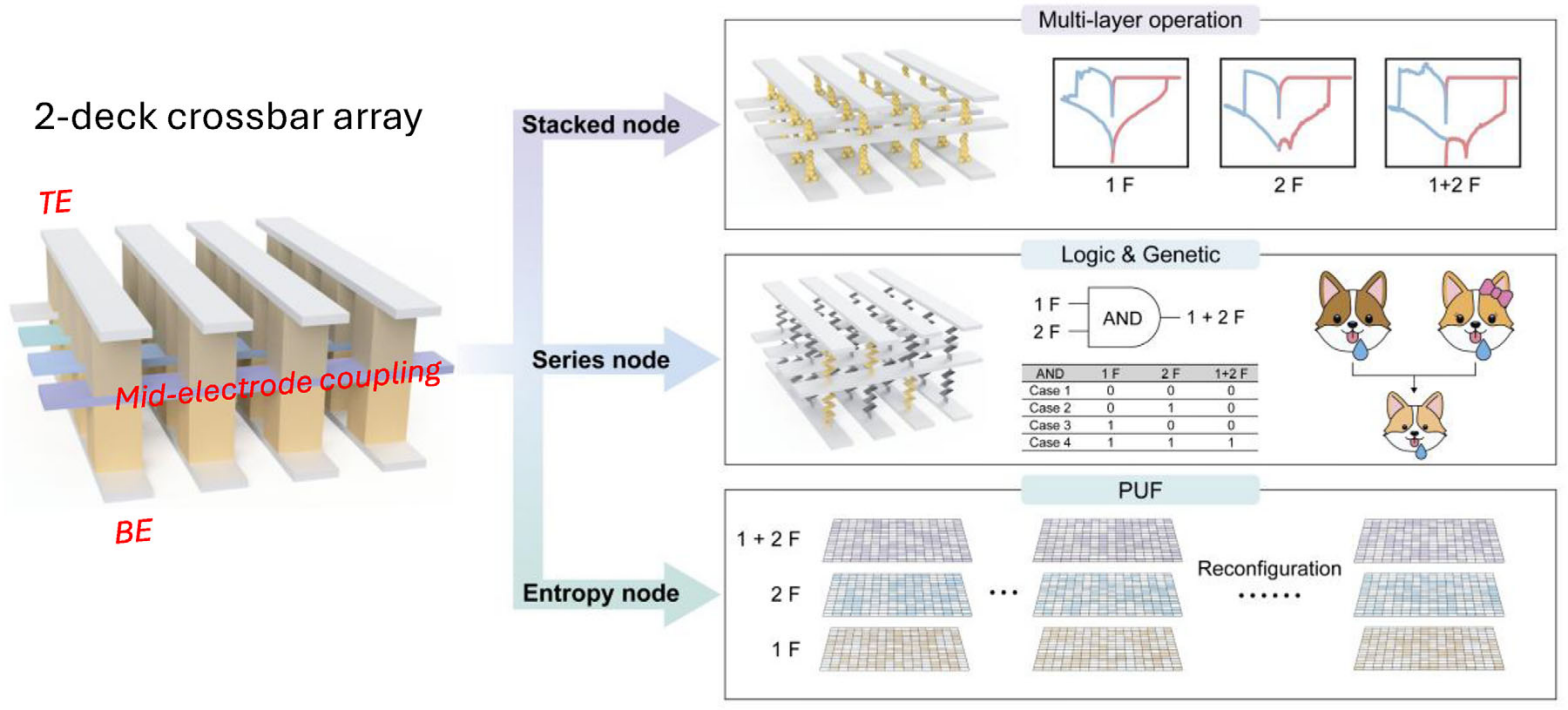
Schematic illustration of the proposed 2 deck crossbar array structure emphasizing the role of the inserted mid electrode. Distinct operation modes depending on application purpose: stacked node for multi‐layer conductance control (1F, 2F, 1F + 2F), series node for logic‐in‐memory and genetic learning functionality, and entropy node for PUF generation and enhanced randomness.

Figure 1 presents the structural concept and operational versatility of the proposed 2 × 16 × 16 stacked RRAM crossbar array incorporating a shared middle electrode (ME). The ME simultaneously serves as the top electrode (TE) of the first switching layer and the bottom electrode (BE) of the second switching layer, enabling compact stacked integration within a single cross‐point footprint. The shared ME plays a dual functional role in the proposed architecture. Structurally, it serves as the common electrode between the two vertically stacked switching layers. Electrically, it acts as a configurable access node that determines the current‐flow path depending on the applied bias condition. By selectively biasing, grounding, or floating the TE, ME, and BE, the device can operate either as an individual switching layer (1F or 2F) or as a serially coupled switching structure (1F + 2F). Consequently, the ME enables both layer‐selective access and controllable interlayer coupling without requiring additional device area or external interconnection circuitry. Depending on the applied bias configuration, the structure operates in three distinct modes: stacked node, series node, and entropy node. In the stacked node configuration, the two switching layers can be addressed independently (1F or 2F) or simultaneously (1F + 2F), allowing three accessible resistance states within the same physical area. In the series node configuration, the two layers form a serial conduction path through the shared ME, enabling current modulation governed by the combined resistance states, which is suitable for logic in memory and genetic learning functionalities [34, 35, 36, 37, 38]. In the entropy node configuration, intrinsic spatial and device‐to‐device current variations across the 16 × 16 array are converted into binary response maps for PUF operation, exploiting the stochastic nature of filamentary switching. The stacked device was fabricated on a thermally oxidized Si substrate. Ti/Pt BE were first patterned, followed by deposition of an ALD‐grown interfacial bilayer and sputtered TaO_x_/TaO_y_ to form the first switching stack. An Al/Pt ME was then defined as a shared electrode between the two layers. To electrically isolate the upper tier, a PECVD SiO_2_ layer was deposited, and contact vias were opened to expose the ME. On this insulated structure, a sputtered SiN layer and the same ALD‐grown interfacial bilayer were sequentially deposited, followed by TaO_x_/TaO_y_ sputtering to construct the second switching stack. Finally, Al/Pt TE electrodes were patterned, completing the 2 × 16 × 16 stacked crossbar array. The overall fabrication flow is summarized in Figure S1. Elemental mapping further confirms the spatial distribution of Pt, Ta, O, Al, Ti, and N, indicating well‐confined switching layers and clear electrode interfaces without noticeable intermixing (Figure S2). Figure S3a,b shows the optical microscopy image of the 2 × 16 × 16 crossbar array and single cell region, confirming well‐defined line patterning with a uniform cell size of 20 × 20 µm^2^. Cross‐sectional TEM analysis clearly reveals two distinct switching layers separated by the shared ME, verifying successful stacked integration. The switching stack employs TaO_x_ as an oxygen reservoir layer and TaO_y_ as a layer intended to moderate filament overgrowth and alleviate current overshoot [39]. However, complete overshoot suppression was not achieved in practice, likely due to localized filament formation dynamics and nonuniform electric field concentration in the stacked geometry. Therefore, external compliance current control was applied during forming and set operations to prevent excessive filament growth and ensure stable resistive switching. This approach allows reliable operation across all three node configurations while maintaining controllable filament evolution in the stacked architecture.

**FIGURE 2:**
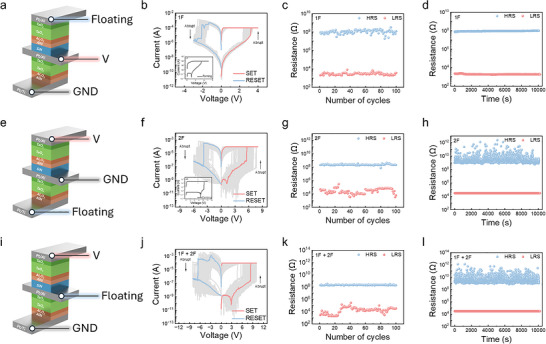
(a) Voltage biasing scheme applied to 1F device, (b) Electroforming and *I–V* characteristics of 1F during the SET and RESET processes, (c) Endurance measurement of 1F after 100 cycles, (d) Retention measurement of 1F, (e) Voltage biasing scheme applied to 2F device, (f) Electroforming and *I–V* characteristics of 2F during the SET and RESET processes, (g) Endurance measurement of 2F after 100 cycles, (h) Retention measurement of 2F, (i) Voltage biasing scheme applied to 1F + 2F device, (j) *I–V* characteristics of 1F + 2F during the SET and RESET processes, (k) Endurance measurement of 1F + 2F after 100 cycles, (l) Retention measurement of 2F.

Figure 2 illustrates the layer‐selective switching behavior of the 2‐deck crossbar structure under different bias configurations, confirming that each resistive layer can be independently accessed within the integrated stack. When the TE was floated, the ME was biased, and the BE was grounded, the applied electric field was confined across the lower switching stack, forcing the current to flow exclusively through the 1F, as illustrated in Figure 2a. The 1F required an initial forming voltage of approximately 5 V and subsequently exhibited stable bipolar switching with SET and RESET voltages of 4 and −3 V over 100 cycles (Figure 2b). The relatively low forming voltage can be attributed to the thin dielectric thickness of the AlN 1.2 and Al_2_O_3_ 2 nm layers combined with the TaO_x_/TaO_y_ stack, which allows sufficient electric field concentration across the active region at moderate bias [40, 41]. The endurance characteristics measured at 0.2 V (Figure 2c) demonstrate consistent resistance modulation over 100 cycles. Furthermore, the retention measurement at 0.2 V over 10^4^ s (Figure 2d) shows stable separation between the HRS and LRS, indicating that filament rupture in the first layer is spatially confined and electrically stable under low read bias. When the TE was biased, the ME was grounded, and the BE was floated, the applied electric field was confined across the 2F, forcing the current to flow exclusively through the 2F (Figure 2e). In this configuration, the device required a significantly higher forming voltage of approximately 15 V, followed by SET and RESET voltages of 8 and −6 V, respectively (Figure 2f). The increased forming voltage is attributed to the combined effects of the additional SiN layer and the increased effective dielectric thickness of the upper switching stack. Compared with the 1F structure, the 2F layer contains an additional 10 nm SiN film beneath the AlN/Al_2_O_3_ and TaO_x_/TaO_y_ layers, resulting in a thicker effective dielectric region across which the electric field must be established. Furthermore, the SiN layer modifies the voltage distribution within the stack and increases the effective barrier for conductive‐filament initiation. Consequently, a higher external bias is required to generate a sufficient local electric field for soft breakdown and subsequent resistive switching. The presence of SiN modifies the electric field distribution and increases the effective barrier for conductive filament initiation, thereby requiring a stronger electric field to trigger soft breakdown. The endurance behavior over 100 cycles measured at 0.2 V is shown in Figure 2g. However, the retention characteristics in Figure 2h reveal that the HRS gradually degrades during the 10^4^ s measurement. This degradation can be related to the dielectric nature of the SiN layer. Due to its relatively high trap density and charge storage capability, trapped carriers within the SiN may slowly redistribute under residual internal fields, facilitating partial reconnection of ruptured conductive paths [42]. As a result, the HRS stability becomes more sensitive to time‐dependent charge relaxation compared to the first layer. When the TE was biased, the ME was floated, and the BE was grounded, the applied electric field was distributed across 1F + 2F, forcing the current to sequentially pass through the 2F, shared ME, and 1F, thereby enabling the coupled 1F + 2F operation, as illustrated in Figure 2i. Notably, when both individual layers were preset, the combined structure did not require an additional forming process and immediately exhibited RESET switching. This behavior originates from the fact that conductive filament pathways had already been established within both the 1F and 2F layers during their individual forming processes. Under the 1F + 2F bias condition, the current sequentially passes through these pre‐existing conductive paths connected through the shared ME, forming a serial conduction path across the entire stacked structure. Consequently, no additional dielectric breakdown is required, as the coupled mode operates through the electrical connection of two previously activated switching layers rather than through the formation of a new conductive filament. The serial configuration showed SET and RESET voltages of 10 and −8 V over 100 cycles (Figure 2j). This intermediate switching voltage, compared to 1F and 2F, reflects voltage division across the two active layers in series. The endurance behavior measured at 0.2 V is presented in Figure 2k. The retention result in Figure 2l indicates that the HRS in the serial configuration is less stable than in the single 1F case. In the serially connected state, an incomplete rupture in either layer can provide a leakage pathway that limits the maximum achievable resistance. Additionally, electric field redistribution between the two stacks may concentrate stress at local weak points, increasing the probability of gradual conductive path recovery during retention. The cell‐to‐cell switching characteristics for nine cells across the 1F, 2F, and 1F + 2F configurations are presented in Figures S4–S6, showing reproducible performance. Overall, the distinct forming voltages and switching characteristics observed under the three bias conditions confirm that the 2‐deck crossbar architecture enables selective and coupled operation of the stacked switching layers within a single integrated structure.

To evaluate synaptic plasticity characteristics under different hierarchical configurations, pulse‐modulated potentiation behaviors were investigated in 1F, 2F, and 1F + 2F. Four modulation schemes were employed: spike amplitude dependent plasticity (SADP), spike width dependent plasticity (SWDP), spike interval dependent plasticity (SRDP), and spike number dependent plasticity (SNDP). For 1F (Figure S7a–d), SADP was examined by modulating the pulse amplitude from 2.7 to 3.0 V while keeping the pulse width (100 µs), pulse interval (100 µs), and pulse number (10) constant. In SWDP, the pulse width was varied from 100 to 400 µs at a fixed amplitude of 2.7 V, with a constant interval (100 µs) and pulse number (10). For SRDP, the pulse interval was modulated from 100 to 400 µs while maintaining a fixed amplitude (2.7 V), pulse width (100 µs), and pulse number (10). In SNDP, the pulse number was increased from 10 to 100 under fixed amplitude (2.7 V), width (100 µs), and interval (100 µs). For 2F (Figure S7e–h), the same modulation schemes were applied with a higher operating voltage. In SADP, the amplitude was varied from 5.7 to 6.0 V while the width (100 µs), interval (100 µs), and pulse number (10) were fixed. In SWDP, the pulse width was modulated from 100 to 400 µs at a constant amplitude of 5.7 V. In SRDP, the pulse interval was varied from 100 to 400 µs under fixed amplitude (5.7 V) and width (100 µs). In SNDP, the pulse number was increased from 10 to 100 while keeping the amplitude (5.7 V), width (100 µs), and interval (100 µs) constant. For 1F + 2F (Figure S7i–l), the operating voltage was further increased. SADP was performed by varying the amplitude from 7.7 to 8.0 V with fixed width (100 µs), interval (100 µs), and pulse number (10). SWDP involved modulation of the pulse width from 100 to 400 µs at a fixed amplitude of 7.7 V. In SRDP, the pulse interval was varied from 100 to 400 µs with constant amplitude (7.7 V) and width (100 µs). In SNDP, the pulse number was increased from 10 to 100 under fixed amplitude (7.7 V), width (100 µs), and interval (100 µs). These results demonstrate that all three configurations support diverse forms of pulse‐modulated synaptic plasticity, while the required operating voltage systematically increases from 1F to 2F and further to the 1F + 2F serial configuration due to the effective resistance contribution of stacked layers.

**FIGURE 3:**
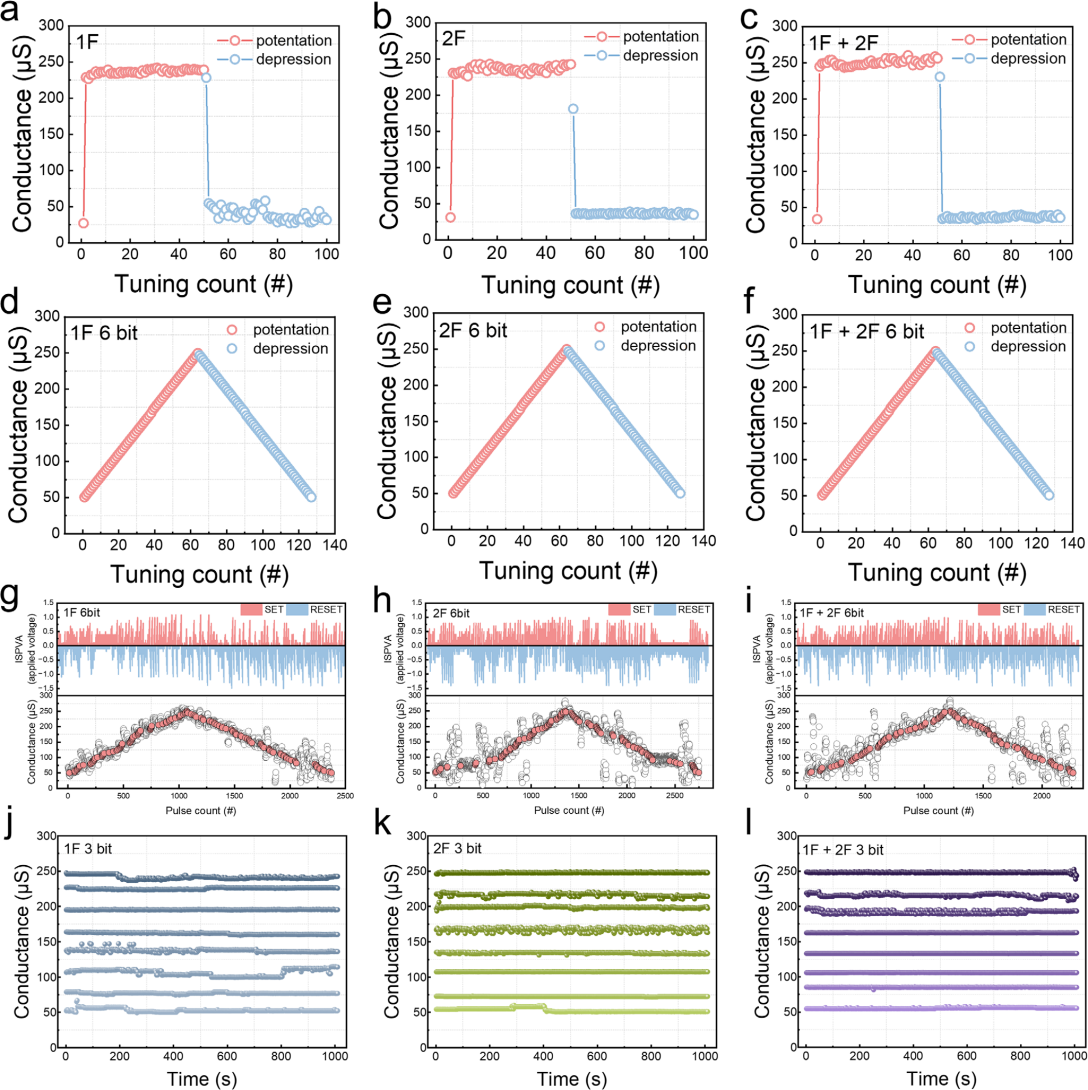
(a) 1F (b) 2F (c) 1F + 2F PD curves by identical pulses, (d) 1F (e) 2F (f) 1F + 2F 6 bit PD curves by ISPVA, Applied voltages and number of pulse attempts for 6 bit states measured in (g) 1F (h) 2F (i) 1F + 2F device, ISPVA retention of 3 bit measured in (j) 1F (k) 2F (l) 1F + 2F device.

Figure 3a–c shows the potentiation and depression (PD) behaviors under fixed amplitude identical pulses. For all configurations, the set and reset pulse widths were fixed at 1 ms, and the read operation was performed at 0.2 V with a pulse width of 100 µs. For 1F (Figure 3a), 50 identical 2 V set pulses and −2 V reset pulses were applied. The conductance transition exhibits abrupt switching characteristics, resulting in highly nonlinear weight updates. Rather than gradual conductance evolution, the device shows sudden transitions once the internal threshold is exceeded, which limits fine analog tunability under simple identical pulse schemes. Similarly, 2F (Figure 3b) was driven by 50 identical 4 V set and −4 V reset pulses. Due to its higher effective switching threshold, larger programming voltages are required compared to 1F. Nevertheless, the conductance update remains abrupt and nonlinear, indicating that the intrinsic filament formation and rupture dynamics dominate the switching behavior [43]. For the serially connected 1F + 2F configuration (Figure 3c), identical 6 V set and −6 V reset pulses were used. Because the applied voltage is distributed across both layers, the total threshold voltage increases, necessitating a higher programming amplitude. The resulting PD characteristics also exhibit strong nonlinearity, reflecting the compounded threshold behavior of the two layers. These results indicate that simple identical pulse programming is insufficient for achieving precise multilevel modulation in all three configurations. To overcome this limitation, an incremental step pulse with verify algorithm (ISPVA) scheme was employed [44, 45]. The detailed programming scheme is illustrated in Figure S8. The improved conductance controllability achieved by the ISPVA scheme can be attributed to the stochastic nature of filament evolution in RRAM devices. Under identical pulse programming, once the local switching threshold is exceeded, filament growth or rupture can occur abruptly, leading to large conductance changes and limited access to intermediate states. In contrast, the gradual increase in programming amplitude employed in ISPVA enables operation closer to the switching threshold, where oxygen vacancy migration and filament evolution occur more progressively. Combined with the intermediate verification process, this approach suppresses excessive filament growth and enables finer conductance modulation across multiple intermediate states. For the ISPVA scheme, all configurations were programmed with a pulse width of 100 µs; the voltage amplitude was increased in 0.1 V steps, and the read operation was performed at 0.2 V with a pulse width of 100 µs. This approach enables controlled traversal of intermediate resistance states by progressively approaching and exceeding local switching thresholds. Under the ISPVA scheme, all three configurations demonstrate significantly improved conductance linearity and multilevel controllability. Up to 6‐bit resolution (64 states) is achieved, as shown in Figure 3d–f. Compared to the abrupt transitions observed under identical pulses, ISPVA allows finer conductance increments by distributing filament evolution across multiple subthreshold and near‐threshold events. Figures S9–S11 show the achievable 1–6 bit conductance states for 1F, 2F, and 1F + 2F under the ISPVA scheme. In all configurations, distinct multilevel states are clearly resolved, demonstrating that incremental amplitude control enables stable and scalable conductance modulation even in the serial 1F + 2F structure. Figures S12–S14 present the number of pulse attempts required to reach each target conductance level for 1F, 2F, and 1F + 2F, respectively. In all configurations, the number of pulse attempts increases as the programmed bit resolution increases. Figure 3j–l shows 3‐bit retention characteristics for 1F, 2F, and 1F + 2F. Distinct conductance levels remain well separated over time, verifying the stability of intermediate resistance states. The retention results further suggest that the device architecture supports reliable multilevel memory operation beyond binary switching. Overall, although the intrinsic switching behavior of 1F, 2F, and 1F + 2F is abrupt and nonlinear under identical pulse driving, the ISPVA strategy effectively linearizes conductance updates and enables high‐resolution multibit operation. Importantly, the 1F + 2F configuration preserves multilevel programmability despite its serially coupled structure, demonstrating that structural stacking does not compromise analog functionality but instead provides an additional design degree of freedom for threshold engineering and programmable state modulation.

**FIGURE 4:**
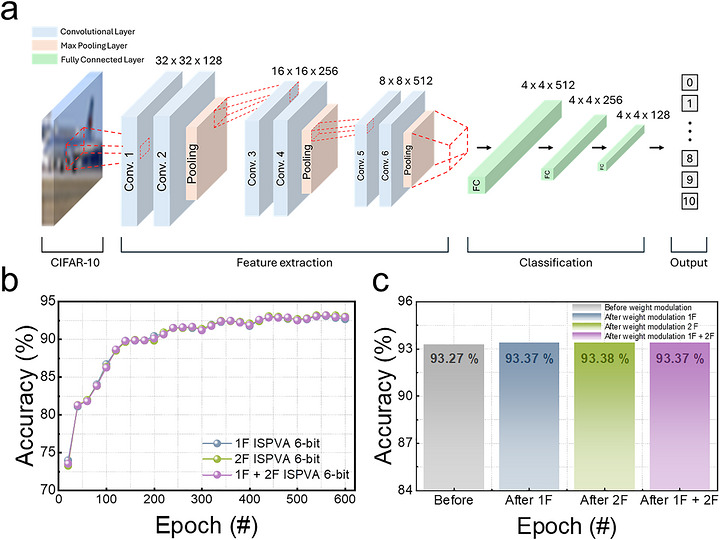
(a) Schematic illustration of the CNN framework utilized to perform image classification on the CIFAR‐10 dataset, (b) Classification accuracy on the CIFAR‐10 dataset plotted against the number of training epochs, (c) Comparison of classification accuracy between the baseline model and models incorporating weight modulation under 1F, 2F, and 1F + 2F conditions.

To verify the applicability of the proposed synaptic device to practical machine learning tasks, a VGG‐based convolutional neural network (CNN) was implemented for CIFAR‐10 image classification, as illustrated in Figure 4a [46, 47]. The architecture consists of sequential convolutional layers for hierarchical feature extraction, interleaved with pooling layers to reduce spatial dimensionality, followed by fully connected (FC) layers for final classification. Nonlinear activation (ReLU) is applied after each convolution to enable complex feature learning. For all configurations (1F, 2F, and 1F + 2F), the synaptic weights were constrained using experimentally obtained ISPVA‐programmed 6‐bit conductance states, ensuring consistent multibit resolution across device structures. The training accuracy recorded every 20 epochs up to 600 epochs is presented in Figure 4b. All configurations exhibit rapid initial improvement followed by stable convergence. The 1F device reaches a peak accuracy of 93.07%, while the 2F and 1F + 2F configurations achieve 93.17% and 93.16%, respectively. After training, the network was instantiated with hardware‐constrained weights by replacing the learned floating‐point parameters with experimentally measured conductance‐derived values obtained from the ISPVA programmed 6‐bit states. The resulting inference accuracy was then compared with the software baseline (Figure 4c). The purely software‐trained model achieved 93.27%, whereas the hardware‐mapped implementations yielded 93.37% for 1F, 93.38% for 2F, and 93.37% for 1F + 2F. The extremely small variation within approximately 0.1% demonstrates that incorporating discrete conductance levels does not degrade classification performance. Instead, the close agreement between baseline and hardware‐mapped results confirms strong consistency between experimentally measured device characteristics and the algorithmic weight distribution. In this framework, CNN training is conducted offline to establish a standardized benchmark on CIFAR‐10, while the hardware contribution is reflected in the readout weight implementation using experimentally obtained multibit conductance states. This approach validates that ISPVA‐enabled synaptic modulation can reliably translate trained software weights into device‐compatible representations without compromising inference accuracy. These results highlight the robustness of the proposed synaptic device against quantization‐induced weight distortion at the system level. Consequently, the demonstrated compatibility with CNN inference confirms its suitability for practical neuromorphic computing applications.

**FIGURE 5:**
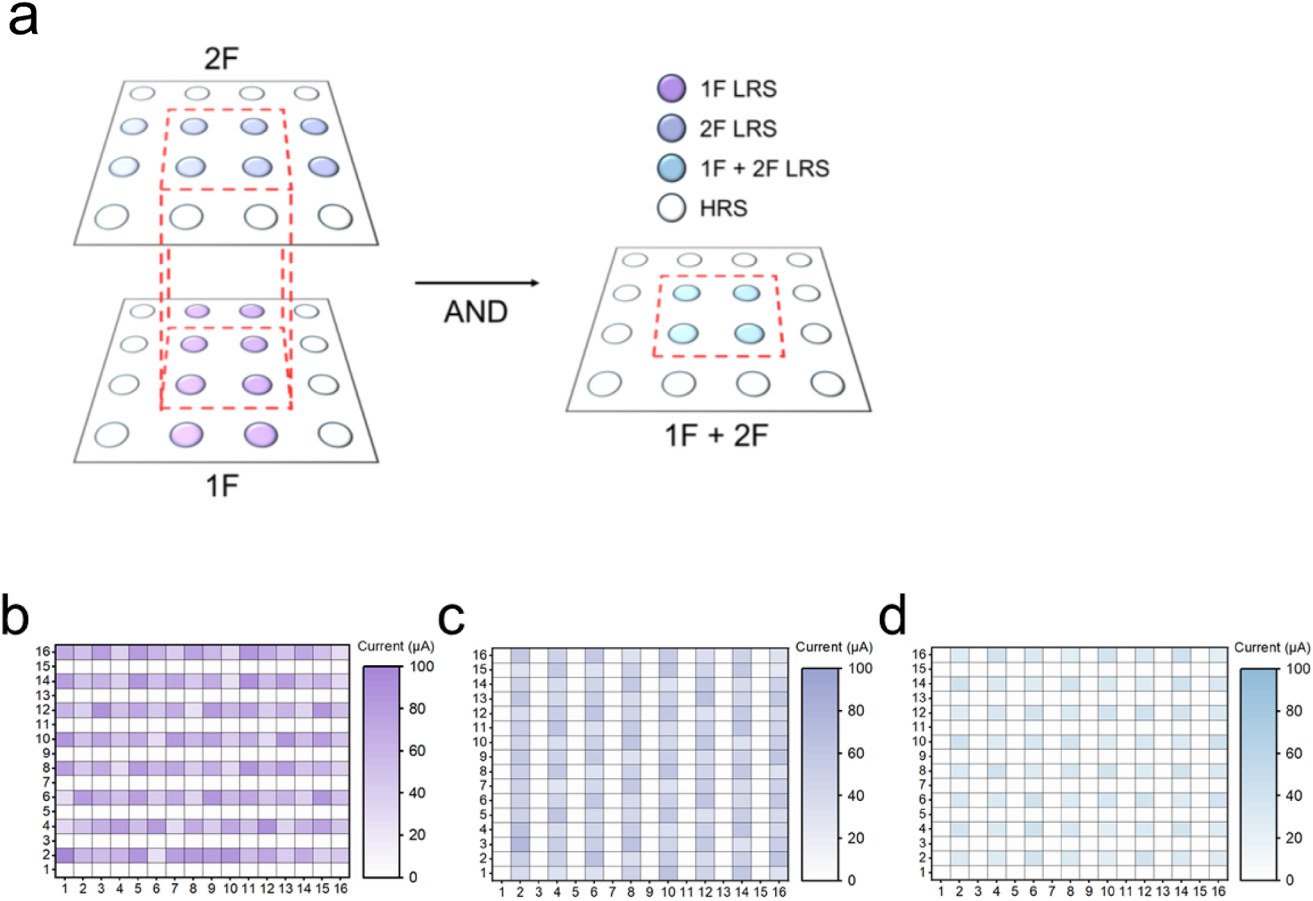
(a) Schematic illustration of logic operation in a 2‐deck crossbar array, 16 × 16 conductance map of (b) 1F, (c) 2F with selective LRS programming, (d) 16 × 16 conductance map of 1F + 2F state reflecting spatial correlation.

Figure 5 demonstrates the logical functionality enabled by the electrically addressable dual‐layer structure. Figure 5a schematically illustrates the AND operation derived from the serial connection of 1F and 2F. Because the current in the 1F + 2F configuration flows sequentially through both layers, the overall resistance state is determined by the combined resistance of the two layers [48, 49]. When both 1F and 2F are in LRS, the total resistance of the serial path is identified as LRS in 1F + 2F. In contrast, if either layer is in HRS, the total resistance is dominated by that layer, resulting in an overall HRS in the serial configuration. This behavior naturally implements an AND logic function based on resistive state overlap. To experimentally verify this operation, 1F and 2F were programmed with distinct patterns in each layer. As shown in Figure 5b, in 1F, LRS was written selectively along the even‐numbered horizontal rows, while the remaining cells were maintained in HRS. In Figures 5c and 2F was programmed independently, where LRS was written along the even‐numbered vertical columns. The two patterns were intentionally designed to intersect at specific cross points within the array. Figure 5d presents the readout of the 1F + 2F configuration. Only the cells where both 1F and 2F were simultaneously programmed to LRS exhibited LRS in the serial read mode. All other positions remained in HRS, even if one of the two layers was in LRS. The resulting pattern clearly corresponds to the logical AND of the two independently programmed layers, confirming that the stacked architecture enables layer‐selective programming and logic‐level state modulation within a single integrated structure. This result highlights that the electrical coupling inherent in the serially connected dual‐layer configuration can be utilized not only for resistive modulation but also for deterministic logic functionality without additional selector devices or external circuitry [50, 51].

As shown in Figure S15, associative learning behavior is observed in both individual layers and the serially coupled structure. For 1F (Figure S15a), a low stimulus of 0.2 V does not exceed the switching threshold and therefore does not induce a conductance change, while a higher voltage of 4 V acts as an unconditioned stimulus that triggers a resistive transition. Through repeated paired stimulation of 4 and 0.2 V, the conductive state is reinforced, allowing the previously subthreshold 0.2 V stimulus to produce a measurable response, analogous to Pavlovian conditioning [52, 53]. A similar behavior is observed in 2F (Figure S15b), where 0.2 V alone cannot induce switching, but repeated pairing with an 8 V stimulus modifies the effective switching condition and enables a response to the low stimulus, demonstrating layer‐specific associative learning [54]. Interestingly, the serially coupled 1F + 2F configuration (Figure S15c) exhibits a response to the 0.2 V stimulus even without additional training. This behavior originates from the electrical coupling between the two layers, where the voltage distribution and resistance modulation across the serial path reduce the effective switching barrier, allowing the low stimulus to generate a measurable current response.

**FIGURE 6:**
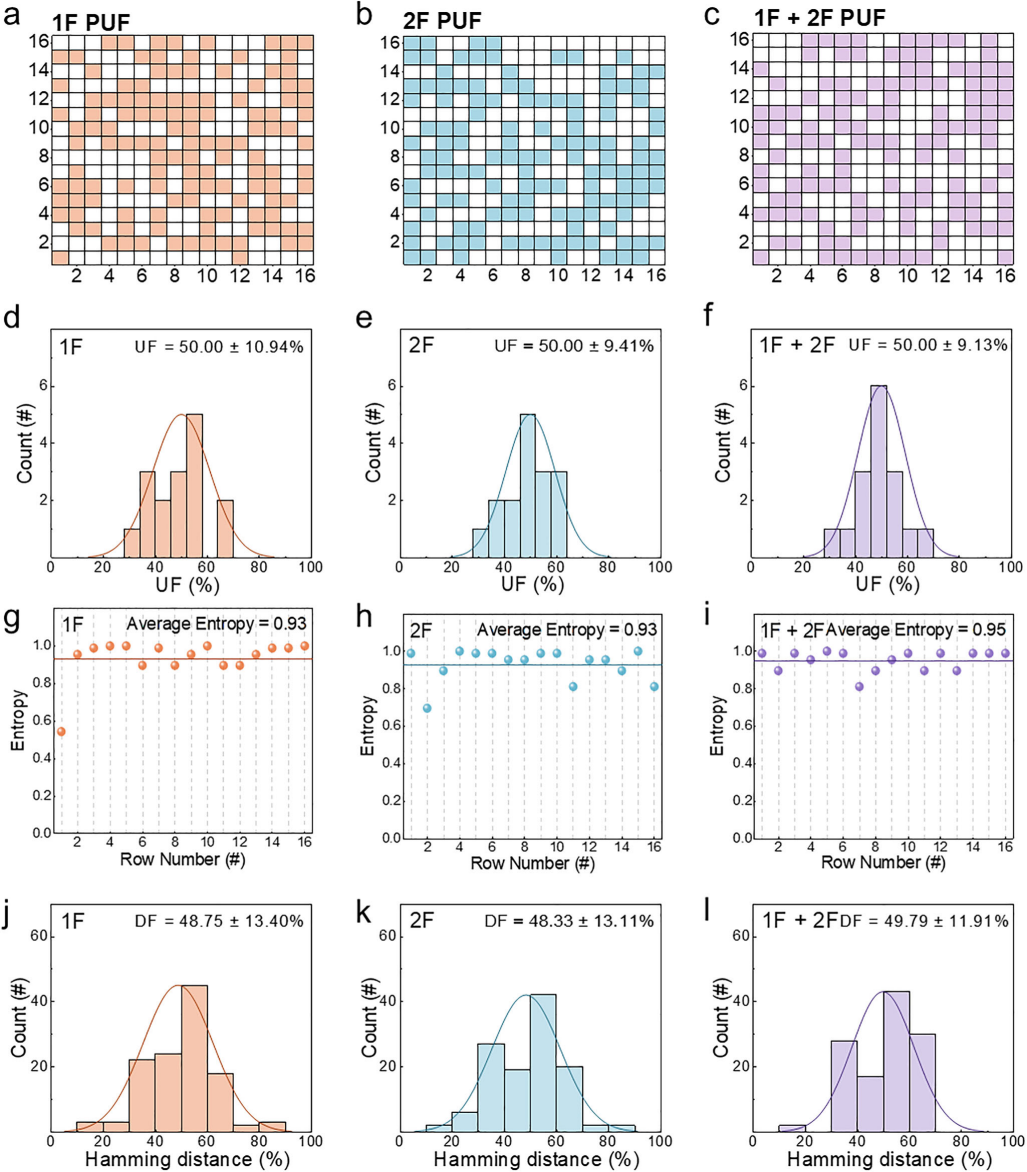
Binary PUF response maps generated by the current readout under three different layer combination conditions: (a) 1F, (b) 2F, and (c) 1F + 2F. Statistical evaluation of the generated PUF responses: row‐wise uniformity factor (UF) distributions for (d) 1F, (e) 2F, and (f) 1F + 2F PUFs. column wise entropy evaluated for (g) 1F, (h) 2F, and (i) 1F + 2F PUFs. Row‐wise Intra‐device Hamming Distance (Intra‐HD) distributions for (j) 1F, (k) 2F, and (l) 1F + 2F PUFs.

In addition to multi‐mode memory, logic, and neuromorphic learning demonstrated above, we further explored whether the intrinsic device‐to‐device and spatial current variations of the proposed two‐tier aligned 2‐deck array can be utilized as an on‐chip security primitive. Binary PUF response maps were generated from the current readout under the same three bias configurations used for hierarchical operation (Figure 6a–c). For each mode, the 16 × 16 current matrix was converted into a binary response by comparing each cell current to an array level reference current (I_ref_), yielding a “1” for I_read > I_ref and a “0” otherwise. This simple conversion allows a direct comparison of randomness and bias across different operating modes without additional circuitry. To quantitatively assess the PUF quality, the uniformity factor (UF), binary entropy (H), and diffuseness factor (DF) were rigorously defined in analytical form [30, 55]. For a given row challenge consisting of *n*binary cells, the response vector is expressed as $r=\{r_{1},r_{2},\text{…},r_{n}\}$, where $r_{i}\in \left\{0,1\right\}$.

The UF is defined as the normalized Hamming weight of the response:

$$UF=\frac{1}{n}\;\sum_{i=1}^{n}r_{i}\times 100\%,$$

where an ideal unbiased PUF yields *UF*  =  50%. The probability of obtaining a "1" in a given row is:

$$p=\frac{1}{n}\;\sum_{i=1}^{n}r_{i},$$

from which the binary entropy is calculated as:


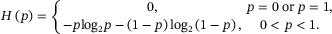




The maximum entropy is reached *H*  =  1when *p*  =  0.5.

To evaluate intra‐device diffuseness, the Hamming distance (HD) between two distinct row responses **r** ^(*a*)^and **r** ^(*b*)^is defined as:

$$HD\left(r^{\left(a\right)},r^{\left(b\right)}\right)=\frac{1}{n}\;\sum_{i=1}^{n}|r_{i}^{\left(a\right)}-r_{i}^{\left(b\right)}|\times 100\%.$$

The DF corresponds to the average intra‐device Hamming distance over all distinct row pairs within the same array, i.e.:

$$DF=\langle HD\left(r^{\left(a\right)},r^{\left(b\right)}\right)\rangle ,$$

where an ideal decorrelated response yields *DF*  =  50%.

As summarized in Figure 6d–l, all three read modes exhibit near‐ideal UF centered at 50%, with UF = 50.00% ± 10.94% (1F), 50.00% ± 9.41% (2F), and 50.00% ± 9.13% (1F + 2F) (Figure 6d–f). The corresponding entropy distributions remain consistently high, yielding average entropy values of 0.93 (1F), 0.93 (2F), and 0.95 (1F + 2F) (Figure 6g–i). Moreover, the DF values derived from the intra‐device Hamming distance are centered close to the ideal 50% level, with DF = 48.75% ± 13.40% (1F), 48.33% ± 13.11% (2F), and 49.79% ± 11.91% (1F + 2F) (Figure 6j–l). These results confirm that all three operating modes satisfy the fundamental statistical requirements for reliable PUF operation, including near‐ideal uniformity, high entropy, and intra‐device decorrelation. Among them, however, the serial 1F + 2F mode exhibits the highest entropy together with comparatively reduced dispersion in both UF and DF, indicating a more stable and statistically balanced bit distribution. This suggests that the series coupled conduction path provides the most favorable operating condition for unbiased bit extraction. We attribute this advantage to the fact that the overall serial current is jointly shaped by both switching layers: the higher‐resistance element naturally suppresses extreme current excursions, while the stacked conduction path effectively mixes stochastic contributions from filament formation/rupture and trap‐mediated transport in each layer, thereby reducing systematic bias and enhancing statistical robustness. Furthermore, this dual‐layer serialization profoundly impacts device‐level variability by convolving the independent probabilistic switching distributions of the two stacks. Instead of being dominated by a single localized microscopic defect, the combined structural variability self‐averages across the vertical cross‐point, effectively tightening the overall distribution and smoothing out tail‐state fluctuations that typically degrade PUF reliability. Finally, we verified the reconfigurability of the PUF responses, which is essential for generating multiple independent keys from the same physical substrate. In this work, reconfiguration was implemented by performing an additional resistive switching cycle in the RRAM array to reinitialize the device states. Specifically, a full reset/set operational cycle—utilizing identical DC voltage sweeps matching the initial electroforming compliance parameters—was applied to completely dissolve the pre‐existing conductive filaments. This reinitialization allows fresh, uncorrelated stochastic filament re‐formation within each cell during the subsequent write cycle. To rigorously evaluate the statistical independence and uniqueness of the resulting maps, the inter‐instance Hamming distance (Inter‐HD) was calculated between all successive reconfigured pairs. The resulting Inter‐HD distribution, centered tightly around the ideal 50% mark, mathematically confirms that each reconfiguration step yields a distinct, cryptographically uncorrelated unique hardware key from the exact same physical substrate. Because filament formation and rupture in RRAM devices are inherently probabilistic processes governed by local defect distributions and ionic migration dynamics, this cycling step effectively reshuffles the cell‐level current distribution, thereby generating a new physically independent PUF instance [56].

**FIGURE 7:**
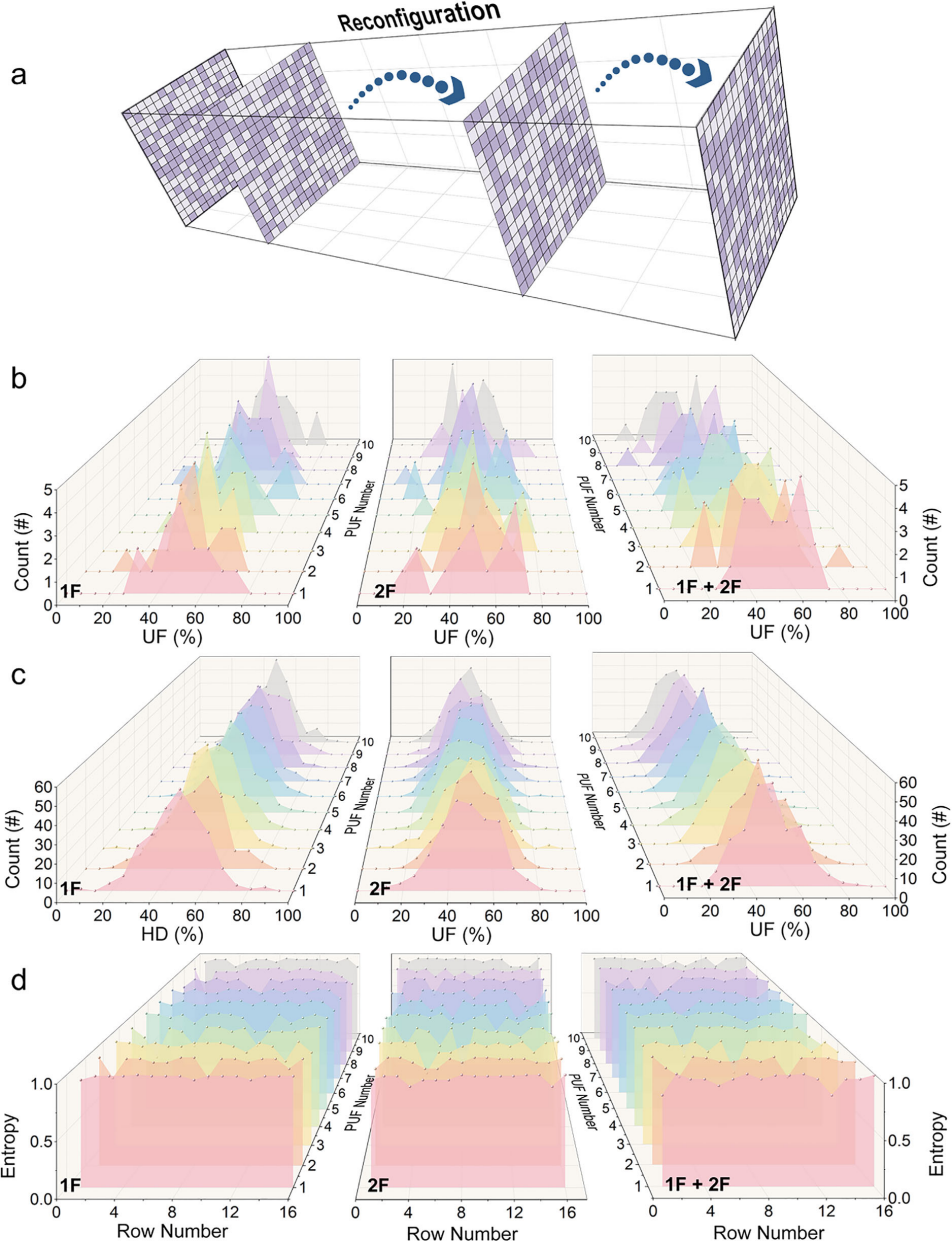
(a) Schematic illustration of the reconfiguration process used to generate multiple PUF instances from the same two‐tier aligned array. 3D statistical representations of reconfigured PUF characteristics for 1F, 2F, and 1F + 2F configurations: (b) row‐wise uniformity factor (UF) distributions obtained from ten independently reconfigured PUFs for each configuration. (c) Hamming distance (HD) distributions calculated between successive PUF instances before and after each reconfiguration step. (d) Column‐wise entropy distributions of the reconfigured PUF responses for the 1F, 2F, and 1F + 2F cases.

As illustrated in Figure 7a, after the reconfiguration cycle, the array current map was reread under the same bias conditions to construct a new binary PUF response. Figure 7b–d present 3D statistical summaries of ten independently reconfigured instances for 1F, 2F, and 1F + 2F. The UF, inter‐instance Hamming distance, and entropy distributions remain consistently centered near their desired values across reconfiguration cycles, indicating that the reconfiguration process produces multiple statistically comparable PUF instances [30, 57] without degrading randomness or introducing systematic bias. Representative 16 × 16 binary response maps of the ten instances are shown in Figure S16, where visibly distinct patterns are obtained after each cycling operation. Taken together, these results demonstrate that the proposed 2‐deck dual‐layer array can serve as a multifunctional hardware primitive, providing not only hierarchical memory/logic/learning operations but also reconfigurable PUF functionality enabled by the intrinsic stochasticity of filamentary switching for intrinsic hardware security.

## 3 Conclusion

In conclusion, a series‐stacked 2‐deck RRAM crossbar array with a shared middle electrode (ME) was demonstrated as a hierarchical multimode hardware platform. The ME enables bias‐selective access to independent single‐layer operation and electrically coupled serial operation within a single‐cell footprint, extending the role of 2‐deck integration beyond simple density scaling. Stable multilevel conductance modulation up to 6‐bit resolution was achieved across all operational modes using the incremental step pulse with verify algorithm (ISPVA) scheme, and system‐level evaluation confirmed that experimentally measured conductance states can be directly mapped to neural network weights without degradation in inference accuracy. Furthermore, the serially coupled configuration inherently supports logic‐in‐memory functionality and robust physical unclonable function generation. These results highlight that series‐coupled 2‐deck RRAM architectures can unify memory, neuromorphic computing, and hardware security in a scalable and reconfigurable platform.

## 4 Experimental Section

The 2‐deck RRAM crossbar array was fabricated on a 4‐inch silicon substrate with a 300 nm‐thick SiO_2_ layer grown by wet thermal oxidation. The bottom electrode was patterned using a negative photoresist and AZ300 developer, followed by deposition of Ti 5 nm and Pt 50 nm by e‐gun evaporation and a lift‐off process to define the bottom electrode (BE) lines. For the first resistive switching stack, a 1 nm‐thick AlN layer was deposited by atomic layer deposition (ALD) at 450°C using trimethylaluminum (TMA) and NH_3_ plasma as the nitrogen source, and a 2 nm‐thick Al_2_O_3_ layer was deposited at the same temperature using TMA and ozone as the precursors. On top of this interfacial bilayer, TaO_x_ 20 nm and TaO_y_ 5 nm were sequentially deposited by sputtering under Ar 20 sccm and O_2_ 0.9/1.1 sccm flow conditions to complete the first switching layer. The middle electrode (ME), composed of Al 10 nm and Pt 25 nm was patterned using the same lithographic process as the bottom electrode and formed by e‐gun evaporation followed by lift‐off. A full area etch was then carried out using ICP at 900 W with CHF_3_ 40 sccm and RF power of 200 W for 35 s in order to expose the BE, where the ME acted as a metal hard mask. To electrically isolate the first and second switching layers, a 50 nm‐thick SiO_2_ layer was deposited by PECVD. Contact holes and contact pads were opened using positive photoresist patterning followed by ICP etching performed at 2200 W ICP power and 100 W RF power under CHF_3_ 60 sccm and Ar 10 sccm for 8 s. The second resistive switching stack was then formed on the isolated structure. A 10 nm‐thick SiN layer was first deposited by sputtering under Ar 20 sccm and N_2_ 5 sccm. ALD layers were stacked in the same conditions and to the same thickness as the first floor. TaO_x_ 30 nm and TaO_y_ 5 nm were then deposited by sputtering under Ar 20 sccm and O_2_ 0.9/1.1 sccm, forming the second switching layer. Finally, the top electrode consisting of Al 10 nm and Pt 25 nm was patterned using the same process as the bottom and middle electrodes, followed by e‐gun evaporation and lift‐off. A final full‐area etch was performed using ICP at 900 W with CHF_3_ 40 sccm and RF power of 200 W for 40 s to expose the bottom and middle electrodes, where the top electrode served as the metal mask. The line widths of the BE, ME, and TE were uniformly defined as 20 × 20 µm^2^.

Electrical measurements were carried out on a probe station using a Keithley 4200‐SCS semiconductor characterization system integrated with a 4225 pulse measurement unit. The switching behavior was evaluated through both quasi‐static DC voltage sweeps and time‐resolved transient measurements. To examine the internal structure and material distribution of the stacked layers, cross‐sectional TEM was employed.

## Author Contributions

**Seungman Park** and **Jaewoo Choi** data curation, formal analysis, and writing – original draft. **Gigon Nam**, **Junsu Yu**, **Donghyun Ryu**, and **Jung‐Kyu Lee** contributed to data curation, resources, software, and writing – review and editing. **Sungjoon Kim** and **Sungjun Kim** were responsible for conceptualization, supervised the project, validated the results, acquired funding, and participated in writing, reviewing, and editing. All authors discussed the results and contributed to the final version of the manuscript.

## Conflicts of Interest

The authors declare no conflicts of interest.

## Supporting information




**Supporting File**: advs76674‐sup‐0001‐SuppMat.docx.

## Data Availability

The data that support the findings of this study are available from the corresponding author upon reasonable request.

## References

[1] D. Ielmini and H.‐S. P. Wong , “In‐Memory Computing With Resistive Switching Devices,” Nature Electronics 1 (2018): 333–343, 10.1038/s41928-018-0092-2.

[2] H. Wang and X. Yan , “Overview of Resistive Random Access Memory (RRAM): Materials, Filament Mechanisms, Performance Optimization, and Prospects,” physica status solidi (RRL)—Rapid Research Letters 13 (2019): 1900073, 10.1002/pssr.201900073.

[3] M. Abedin , N. Gong , K. Beckmann , et al., “Material to System‐Level Benchmarking of CMOS‐Integrated RRAM With Ultra‐Fast Switching For Low Power On‐Chip Learning,” Scientific Reports 13 (2023): 14963, 10.1038/s41598-023-42214-x. 37697024 PMC10495451

[4] A. Wali and S. Das , “Two‐Dimensional Memtransistors for Non‐Von Neumann Computing: Progress and Challenges,” Advanced Functional Materials 34 (2024): 2308129, 10.1002/adfm.202308129.

[5] S. Shahrabadi , “Resistive Random Access Memory (RRAM) 1960–2025: Review,” IEEE Electron Devices Reviews 2: 215–252, 10.1109/EDR.2025.3642784.

[6] N. Andreeva , A. Ivanov , and A. Petrov , “Multilevel Resistive Switching in TiO_2_/Al_2_O_3_ Bilayers At Low Temperature,” AIP Advances 8 (2018): 025208.

[7] H. So , H. Ji , S. Kim , and S. Kim , “Sophisticated Conductance Control and Multiple Synapse Functions in TiO_2_ ‐Based Multistack‐Layer Crossbar Array Memristor for High‐Performance Neuromorphic Systems,” Advanced Functional Materials 34 (2024): 2405544, 10.1002/adfm.202405544.

[8] Y. Byun , G. Kim , S. Kim , and S. Kim , “Reset‐Dominant Accurate Synaptic Weight Mapping In Passive Memristor Arrays For Energy‐Efficient Spiking Neural Networks,” Nano Energy 142 (2025): 111261, 10.1016/j.nanoen.2025.111261.

[9] A. Grossi , E. Perez , C. Zambelli , et al., “Impact Of The Precursor Chemistry And Process Conditions On The Cell‐To‐Cell Variability in 1T‐1R Based HfO_2_ RRAM Devices,” Scientific Reports 8 (2018): 11160, 10.1038/s41598-018-29548-7. 30042433 PMC6057879

[10] J. Park , J. Lee , S. Youn , and H. Kim , “Step‐Efficient Parallel Implementation of *n*‐bit Full Adders Using Stateful Logic in Memristor Crossbar Arrays,” Advanced Intelligent System 8 (2026): 202501001.

[11] S. Youn , K. Kim , J. Park , and H. Kim , “Energy‐Efficient Ising Solver Implementations In Forming‐Free Memristor Crossbar Arrays For Combinatorial Optimization Problems,” Nano Energy 148 (2026): 111633, 10.1016/j.nanoen.2025.111633.

[12] J. Park , A. Kumar , Y. Zhou , et al., “Multi‐Level, Forming And Filament Free, Bulk Switching Trilayer RRAM for Neuromorphic Computing At The Edge,” Nature Communications 15 (2024): 3492, 10.1038/s41467-024-46682-1. PMC11045755 38664381

[13] U. Byun , H. Na , and S. Kim , “Universal Neuromorphic Element: NbO_x_ Memristor With Co‐Existing Volatile, Non‐Volatile, and Threshold Switching,” Advanced Functional Materials 36 (2026): 19431, 10.1002/adfm.202519431.

[14] M. Ismail , D. Kim , E. Lim , et al., “Exploration of Analog Synaptic Plasticity and Convolutional Neural Network Simulation in Bilayer TiO x N y /SnO x Memristor for Neuromorphic Systems,” ACS Materials Letters 6 (2024): 3514–3522, 10.1021/acsmaterialslett.4c00406.

[15] M. Hu , H. Li , Y. Chen , Q. Wu , G. S. Rose , and R. W. Linderman , “Memristor Crossbar‐Based Neuromorphic Computing System: A Case Study,” IEEE Transactions on Neural Networks and Learning Systems 25 (2014): 1864–1878, 10.1109/TNNLS.2013.2296777. 25291739

[16] D. Kudithipudi , C. Schuman , C. M. Vineyard , et al., “Neuromorphic Computing At Scale,” Nature 637 (2025): 801–812, 10.1038/s41586-024-08253-8. 39843589

[17] X. Zhang , A. Huang , Q. Hu , Z. Xiao , and P. K. Chu , “Neuromorphic Computing With Memristor Crossbar,” Physica Status Solidi (a) 215 (2018): 1700875, 10.1002/pssa.201700875.

[18] Y. Du , J. Tang , Y. Li , et al., “Monolithic 3D Integration of Analog RRAM‐Based Computing‐in‐Memory and Sensor for Energy‐Efficient Near‐Sensor Computing,” Advanced Materials 36 (2024): 2302658, 10.1002/adma.202302658. 37652463

[19] J. Y. Seok , S. J. Song , J. H. Yoon , et al., “A Review of Three‐Dimensional Resistive Switching Cross‐Bar Array Memories From the Integration and Materials Property Points of View,” Advanced Functional Materials 24 (2014): 5316–5339, 10.1002/adfm.201303520.

[20] R. An , Y. Li , and J. Tang , “A Hybrid Computing‐In‐Memory Architecture by Monolithic 3D Integration of BEOL CNT/IGZO‐based CFET Logic and Analog RRAM,” in 2022 International Electron Devices Meeting (IEDM) (IEEE, 2022).

[21] S.‐P. Sing , Y.‐C. Wang , W.‐H. Lin , et al., “A New High Density 3D Stackable Via RRAM for Computing‐in‐Memory SOC Applications,” IEEE Transactions on Electron Devices 71 (2024): 2399–2403, 10.1109/TED.2024.3367661.

[22] Y. Choi , S. Oh , C. Qian , J.‐H. Park , and J. H. Cho , “Vertical Organic Synapse Expandable to 3D Crossbar Array,” Nature Communications 11 (2020): 4595, 10.1038/s41467-020-17850-w. PMC7490352 32929064

[23] V. Manouras , S. Stathopoulos , A. Serb , and T. Prodromakis , “Selectively Biased Tri‐Terminal Vertically‐Integrated Memristor Configuration,” Scientific Reports 12 (2022): 10467, 10.1038/s41598-022-14462-w. 35729336 PMC9213395

[24] A. A. Sharma , M. Noman , M. Abdelmoula , M. Skowronski , and J. A. Bain , “Electronic Instabilities Leading to Electroformation of Binary Metal Oxide‐Based Resistive Switches,” Advanced Functional Materials 24 (2014): 5522–5529, 10.1002/adfm.201400461.

[25] A. Subramanian , N. Tiwale , K. Kisslinger , and C.‐Y. Nam , “Reduced Stochastic Resistive Switching in Organic‐Inorganic Hybrid Memristors by Vapor‐Phase Infiltration,” Advanced Electronic Materials 8 (2022): 2200172, 10.1002/aelm.202200172.

[26] L. Peddaboina , K. Agrawal , P. Kumar , G. Hegde , O. Badami , and S. Bhattacharjee , “A Variability‐Aware Behavioral Model of Monolayer MoS_2_ RRAM for Tunable Stochastic Sources,” Advanced Theory and Simulations 8 (2025): 2401235, 10.1002/adts.202401235.

[27] S. Yu , X. Guan , and H.‐S. P. Wong , “On the Stochastic Nature Of Resistive Switching In Metal Oxide RRAM: Physical Modeling, Monte Carlo Simulation, And Experimental Characterization,” IEEE International Electron Devices Meeting (2011): 17.3.1–17.3.4, 10.1109/IEDM.2011.6131572.

[28] C.‐J. Beak , S. Park , J. Park , H.‐L. Park , and S.‐H. Lee , “Stochastic Resistive Switching In Memristors As Physically Unclonable Functions,” Journal of Physics D: Applied Physics 58 (2025): 443002, 10.1088/1361-6463/ae09b8.

[29] J. Park and H. Kim , “Physical Unclonable Function With 3D Stacked Memristor Crossbar Array Using Self‐Differential Pair,” ACS Nano 19 (2025): 28135–28145, 10.1021/acsnano.4c18621. 40719704

[30] S. Park , H. Na , J. Choi , et al., “Dual‐State Conversion For High‐Entropy And Reconfigurable Resistive Memory‐Based Physically Unclonable Functions,” Journal of Materials Science & Technology 266 (2026): 38–47, 10.1016/j.jmst.2025.11.025.

[31] F. Zahoor , U. I. Bature , A. Nisar , A. Alzahrani , H. Abbas , and F. Bashir , “A Comprehensive Review on Physical Unclonable Functions Based on Resistive Random Access Memory,” ACS Applied Electronic Materials 7 (2025): 6215–6242, 10.1021/acsaelm.5c00504.

[32] H. M. Ibrahim , H. Abunahla , B. Mohammad , and H. AlKhzaimi , “Memristor‐Based PUF for Lightweight Cryptographic Randomness,” Scientific Reports 12 (2022): 8633, 10.1038/s41598-022-11240-6. 35606367 PMC9126908

[33] R. Carboni and D. Ielmini , “Stochastic Memory Devices for Security and Computing,” Advanced Electronic Materials 5 (2019): 1900198.

[34] X. Xiong , J. Kang , Q. Hu , et al., “Reconfigurable Logic‐in‐Memory and Multilingual Artificial Synapses Based on 2D Heterostructures,” Advanced Functional Materials 30 (2020): 1909645, 10.1002/adfm.201909645.

[35] F. Wei , X. Cui , and X. Cui , “An Improved iMemComp OR Gate and its Applications in Logic Circuits,” IEEE Journal of the Electron Devices Society 8 (2020): 57–61, 10.1109/JEDS.2019.2962822.

[36] D. Kim , S. Kim , and S. Kim , “Logic‐In‐Memory Application of CMOS Compatible Silicon Nitride Memristor,” Chaos, Solitons & Fractals 153 (2021): 111540, 10.1016/j.chaos.2021.111540.

[37] T. D. Dongale , G. U. Kamble , D. Y. Kang , S. S. Kundale , H.‐M. An , and T. G. Kim , “Recent Progress in Selector and Self‐Rectifying Devices for Resistive Random‐Access Memory Application,” physica status solidi (RRL)—Rapid Research Letters 15 (2021): 2100199, 10.1002/pssr.202100199.

[38] B. Yan , B. Li , X. Qiao , et al., “Resistive Memory‐Based In‐Memory Computing: From Device and Large‐Scale Integration System Perspectives,” Advanced Intelligent Systems 1 (2019): 1900068, 10.1002/aisy.201900068.

[39] S. Kim , H. Ji , S. Kim , and W. Y. Choi , “Enhanced Reliability and Self‐Compliance of Synaptic Arrays for Multibit Encoded Neuromorphic Systems,” Advanced Electronic Materials 11 (2025): 2400282, 10.1002/aelm.202400282.

[40] S. Kim , K. Hong , H. Kim , M.‐H. Kim , and W. Y. Choi , “Overshoot‐Suppressed Memristor Array With AlN Oxygen Barrier for Low‐Power Operation in the Intelligent Neuromorphic Systems,” Advanced Intelligent Systems 6 (2024): 2300797, 10.1002/aisy.202300797.

[41] S. Kim , K. Park , K. Hong , et al., “Overshoot‐Suppressed Memristor Crossbar Array With High Yield by AlO x Oxidation for Neuromorphic System,” Advanced Materials Technologies 9 (2024): 2400063, 10.1002/admt.202400063.

[42] S. Kim and B.‐G. Park , “Nonlinear and Multilevel Resistive Switching Memory in Ni/Si_3_N_4_/Al_2_O_3_/TiN Structures,” Applied Physics Letters 108 (2016): 212103, 10.1063/1.4952719.

[43] S. Menzel , M. Waters , A. Marchewka , U. Böttger , R. Dittmann , and R. Waser , “Origin of the Ultra‐Nonlinear Switching Kinetics in Oxide‐Based Resistive Switches,” Advanced Functional Materials 21 (2011): 4487–4492, 10.1002/adfm.201101117.

[44] H. Na and S. Kim , “Enhanced Reliability and Controllability in Filamentary Oxide‐Based 3D Vertical Structured Resistive Memory With Pulse Scheme Algorithm for Versatile Neuromorphic Applications,” Advanced Functional Materials 35 (2025): 2500956, 10.1002/adfm.202500956.

[45] M. Noh , Y. Byun , G. Kim , J. Park , S. Kim , and S. Kim , “Array‐Integrated Memristor With an Interference‐Suppressed Pulse Scheme for Multibit Neuromorphic and Edge Computing,” ACS Applied Electronic Materials 7 (2025): 8211–8226, 10.1021/acsaelm.5c01300.

[46] Q. Zheng , M. Yang , X. Tian , N. Jiang , and D. Wang , “A Full Stage Data Augmentation Method In Deep Convolutional Neural Network For Natural Image Classification,” Discrete Dynamics in Nature and Society 2020 (2020): 4706576.

[47] Z. Li , F. Liu , W. Yang , S. Peng , and J. Zhou , “A Survey of Convolutional Neural Networks: Analysis, Applications, and Prospects,” IEEE Transactions on Neural Networks and Learning Systems 33 (2022): 6999–7019, 10.1109/TNNLS.2021.3084827. 34111009

[48] Z. Sun , E. Ambrosi , A. Bricalli , and D. Ielmini , “Logic Computing With Stateful Neural Networks of Resistive Switches,” Advanced Materials 30 (2018): 1802554, 10.1002/adma.201802554. 30079525

[49] I. Vourkas and G. C. Sirakoulis , “Emerging Memristor‐Based Logic Circuit Design Approaches: A Review,” IEEE Circuits and Systems Magazine 16 (2016): 15–30, 10.1109/MCAS.2016.2583673.

[50] T.‐Y. Wang , J.‐L. Meng , L. Chen , et al., “Flexible 3D Memristor Array For Binary Storage And Multi‐States Neuromorphic Computing Applications,” InfoMat 3 (2021): 212–221, 10.1002/inf2.12158.

[51] P. Sun , N. Lu , L. Li , et al., “Thermal Crosstalk in 3‐Dimensional RRAM Crossbar Array,” Scientific Reports 5 (2015): 13504, 10.1038/srep13504. 26310537 PMC4550907

[52] M. Prezioso , F. Merrikh‐Bayat , B. D. Hoskins , G. C. Adam , K. K. Likharev , and D. B. Strukov , “Training And Operation Of An Integrated Neuromorphic Network Based On Metal‐Oxide Memristors,” Nature 521 (2015): 61–64, 10.1038/nature14441. 25951284

[53] M. Ziegler , R. Soni , T. Patelczyk , et al., “An Electronic Version of Pavlov's Dog,” Advanced Functional Materials 22 (2012): 2744–2749, 10.1002/adfm.201200244.

[54] Z. Wang , S. Joshi , S. E. Savel'ev , et al., “Memristors With Diffusive Dynamics As Synaptic Emulators For Neuromorphic Computing,” Nature Materials 16 (2017): 101–108, 10.1038/nmat4756. 27669052

[55] Y. Cao , W. Liu , L. Qin , et al., “Entropy Sources Based On Silicon Chips: True Random Number Generator And Physical Unclonable Function,” Entropy 24 (2022): 1566. 36359655 10.3390/e24111566 PMC9689501

[56] R. Waser and M. Aono , “Nanoionics‐Based Resistive Switching Memories,” Nature Materials 6 (2007): 833–840, 10.1038/nmat2023. 17972938

[57] A. Chen , “Reconfigurable Physical Unclonable Function Based On Probabilistic Switching of RRAM,” Electronics Letters 51 (2015): 615–617, 10.1049/el.2014.4375.

