# Supplementary material for: Hierarchical Multi‐Mode Computing in Interlayer‐Coupled 3D RRAM Crossbar Arrays
Source: Adv Sci (Weinh). 2026 Jul 30;13(58):e76674. doi: 10.1002/advs.76674 (PMC13421840; doi:10.1002/advs.76674)
Supplement: Supplementary file 1 [file ADVS-13-e76674-s001.docx]

**Hierarchical Multi-Mode Computing in Interlayer-Coupled 3D RRAM Crossbar Arrays**

*Seungman Park^1†^, Jaewoo Choi^1†^, Gigon Nam^1^, Junsu Yu^2^, Donghyun Ryu^3^, Jung-Kyu Lee^4^, Sungjoon Kim^5*^, Sungjun Kim^1*^*

^1^Division of Electronics and Electrical Engineering, Dongguk University, Seoul 04620, Republic of Korea

^2^School of Electrical and Computer Engineering, Georgia Institute of Technology, Atlanta, GA, USA

^3^Electrical Engineering and Computer Sciences, University of California, Berkeley, CA, USA

^4^Department of Semiconductor Engineering, Gyeongsang National University, Jinju, Gyeongnam 52828, Republic of Korea

^5^Department of AI Semiconductor Engineering, Korea University, Sejong 30019, Republic of Korea

**Supplementary Information**

**Figure S1.** Schematic illustrations of each process.

**Figure S2.** Atomic percentage and EDS image of Pt, Ta, O, Al, Ti, N.

**Figure S3.** a) Top-view CD-SEM image of crossbar array, b) Top-view CD-SEM image of single cell and cross-sectional TEM image.

**Figure S4.** Cell-to-cell variation of 1F device.

**Figure S5.** Cell-to-cell variation of 2F device.

**Figure S6.** Cell-to-cell variation of 1F + 2F device.

**Figure S7.** Synaptic weight modulation under various pulse schemes a) 1F SADP b) 1F SWDP c) 1F SRDP d) 1F SNDP e) 2F SADP f) 2F SWDP g) 2F SRDP h) SNDP i) 1F + 2F SADP j) 1F + 2F SWDP k) 1F + 2F SRDP l) 1F + 2F SNDP.

**Figure S8.** a) Schematic illustration of the Incremental step pulse with Verify Algorithm (ISPVA) programming procedure for gradual conductance tuning toward the target state. b) ISPVA programming scheme with intermediate verify process for accurate conductance control.

**Figure S9.** PD curves obtained by ISPVA in the 1F device. a) 1 bit, b) 2 bit, c) 3 bit, d) 4 bit, e) 5 bit, f) 6 bit.

**Figure S10.** PD curves obtained by ISPVA in the 2F device. a) 1 bit, b) 2 bit, c) 3 bit, d) 4 bit, e) 5 bit, f) 6 bit.

**Figure S11.** PD curves obtained by ISPVA in the 1F + 2F device. a) 1 bit, b) 2 bit, c) 3 bit, d) 4 bit, e) 5 bit, f) 6 bit.

**Figure S12.** **.** Number of pulse attempts for multi-bit states measured in 1F device. a) 1 bit, b) 2 bit, c) 3 bit, d) 4 bit, e) 5 bit, f) 6 bit.

**Figure S13.** Number of pulse attempts for multi-bit states measured in 2F device. a) 1 bit, b) 2 bit, c) 3 bit, d) 4 bit, e) 5 bit, f) 6 bit.

**Figure S14.** Number of pulse attempts for multi-bit states measured in 1F + 2F device. a) 1 bit, b) 2 bit, c) 3 bit, d) 4 bit, e) 5 bit, f) 6 bit.

**Figure S15.** a) Pavlovian conditioning response in the father dog (1F), b) Pavlovian conditioning response in mother dog (2F), c) Pavlovian conditioning response in the offspring dog (genetic learning, 1F + 2F) via combined 1F-2F layers with series-resistance-induced threshold crossing, simultaneous LRS states, and a conditioned bell response without explicit training.

**Figure S16.** Binary PUF response maps (16×16) of ten reconfigured PUF instances for each of a) 1F, b) 2F, and c) 1F + 2F.

**Figure S17.** a) Array configuration showing selected, half-selected, and unselected cells in the presence of sneak-path current, b) Read current of the selected cell, c) Average read current of half-selected cells, d) Average read current of unselected cells.
